# Overview of the SACLA facility

**DOI:** 10.1107/S1600577515004658

**Published:** 2015-04-16

**Authors:** Makina Yabashi, Hitoshi Tanaka, Tetsuya Ishikawa

**Affiliations:** a RIKEN SPring-8 Center, Kouto 1-1-1, Sayo, Hyogo 679-5148, Japan

**Keywords:** X-ray free-electron laser, beamline instrumentation, X-ray optics, ultrafast X-ray science, quantum X-ray optics

## Abstract

In March 2012, SACLA started user operations of the first compact X-ray free-electron laser (XFEL) facility. SACLA has been routinely providing users with stable XFEL light over a wide photon energy range from 4 to 15 keV and an ultrafast pulse duration below 10 fs. The facility supports experimental activities in broad fields by offering high-quality X-ray optics and diagnostics, as well as reliable multiport charge-coupled-device detectors, with flexible experimental configurations. A two-stage X-ray focusing system was developed that enables the highest intensity of 10^20^ W cm^−2^. Key scientific results published in 2013 and 2014 in diverse fields are reviewed. The main experimental systems developed for these applications are summarized. A perspective on the facility upgrade is presented.

## Abstract 2

An overview of SACLA operating as a user facility is presented. An updated status of the light source and the beamline is summarized. Key scientific achievements reported in 2013 and 2014 and the main experimental systems are reviewed. A perspective of the facility upgrade is given.

## 1. Introduction   

In June 2011, SPring-8 Angstrom Compact free-electron LAser (SACLA) in SPring-8, Harima, Japan (Ishikawa *et al.*, 2012 ▶), achieved first lasing at 10 keV. User operations commenced the following year, in March 2012. SACLA is the second X-ray free-electron laser (XFEL) facility in operation after the Linac Coherent Light Source (LCLS) in the USA (Emma *et al.*, 2010 ▶), and the first compact XFEL facility in the world.

The idea for the compact XFEL light source was initially conceived by one of the authors (TI) and H. Kitamura in the late 1990s. A key technology is the in-vacuum undulator, which has been widely used in the SPring-8 storage ring as a standard for undulators to produce hard X-rays. The device can be operated with a very small gap between magnetic arrays inside a vacuum vessel to produce a higher magnetic field as compared with those achieved using conventional undulators. This characteristic enables practical operation of shorter-period undulators to generate shorter-wavelength radiation even with a moderate electron-beam energy, as seen in the formula
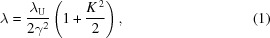
where λ, λ_U_, γ and *K* represent the radiation wavelength, the undulator period length, the electron beam energy and the undulator parameter, respectively. The reduced electron beam energy allows the length of the linear accelerator, which is mostly proportional to the electron beam energy, to be shortened.

In 2001, we started an R&D program at RIKEN for the development of elementary technologies supporting the compact XFEL sources based on a self-amplified spontaneous emission (SASE) scheme. T. Shintake, who developed a 5.7 GHz C-band accelerator system at KEK that has a higher acceleration gradient than those in conventional 2.8 GHz S-band systems, joined the team seeking to utilize the C-band system to achieve a more compact XFEL source, as well as to develop a unique injector system. The injector system is based on a classical thermionic electron gun followed by a velocity bunching system to generate a low-emittance, short-bunch and high-density electron beam.

Following R&D on key components, we constructed a prototype free-electron laser (FEL) working in the extreme ultra-violet (EUV) region in 2005 to confirm the feasibility of the compact XFEL system. This machine, named the SCSS test accelerator (SCSS), had an electron-beam energy of 250 MeV. After an intensive commissioning period led by one of the authors (HT), we observed first lasing with the SASE scheme at λ = 49 nm in 2006 (Shintake *et al.*, 2008 ▶). The SCSS was utilized for basic R&D of the accelerator and beamline systems, studies on HHG seeding (Lambert *et al.*, 2008 ▶; Togashi *et al.*, 2011 ▶), and photon science using an EUV-FEL light, in close collaboration with a number of research groups (Yabashi *et al.*, 2013 ▶). After completing these critical tasks, the SCSS was decommissioned in 2013.

In parallel to these accelerator studies, we performed R&D on X-ray optics to fully utilize coherent X-rays from an XFEL light source (Yabashi *et al.*, 2014 ▶). For this purpose, we exploited two unique X-ray beamlines at SPring-8: a 1 km beamline BL29XU (Ishikawa *et al.*, 2001 ▶) and a 25 m undulator beamline 19LXU (Hara *et al.*, 2002 ▶). Using these beamlines, we successfully developed high-quality optics for coherent X-ray applications, including ultraprecise reflective mirrors (Yamauchi, 2015 ▶), speckle-free Be windows and diamond thin films (Goto *et al.*, 2007 ▶), a dispersive X-ray spectrometer (Yabashi *et al.*, 2006 ▶), thin Bragg crystals (Osaka *et al.*, 2013 ▶; Feng *et al.*, 2012 ▶) and a transmissive beam monitor (Tono *et al.*, 2011 ▶). In particular, we significantly improved the quality of the mirrors in close collaboration with Osaka University, based on elastic emission machining (EEM) technology (Yamauchi *et al.*, 2002 ▶) and precise metrologies, as described by Yamauchi (2015 ▶).

Following these achievements, the Japanese government approved the project for constructing the compact XFEL facility, later named SACLA, as one of the key national technologies in 2006. The facility would have a total length of 700 m. N. Kumagai, who led the construction project of the SPring-8 storage ring, was appointed to lead the accelerator construction of SACLA. Construction progressed smoothly to completion in March 2011. We also completed construction of the SACLA–SPring-8 experimental facility in 2012, which provides a unique opportunity for simultaneous utilization of XFEL with synchrotron radiation.

We started fine-tuning of SACLA in April 2011. To achieve lasing, one needs to satisfy a sufficient overlap of the electron beam with the photon beam in the undulators over 100 m with an extreme accuracy of microradians. For this purpose, we developed a unique method for ‘photon-based alignment’ (Tanaka *et al.*, 2012 ▶). Here, the spontaneous emission of X-rays from each undulator segment was introduced to the double-crystal monochromator (DCM) and imaged on a multiport charge coupled device (MPCCD) detector (Kameshima *et al.*, 2014 ▶), which was later replaced by a phosphor-coupled multichannel plate (MCP), installed in the photon beamline (Tono *et al.*, 2013 ▶). Monitoring of the central cone of the X-ray beam allowed us to determine the electron beam trajectory with a sufficient precision. The synergetic use of the accelerator and beamline, though they have generally been operated independently in conventional synchrotron light sources, enabled us to accomplish the first X-ray lasing at a wavelength of 0.12 nm in June, only two months after starting the fine-tuning.

In March 2012, we opened operations for international users. The JASRI User Office operated reviews of proposals, similar to SPring-8 (http://sacla.xfel.jp/?p=190&lang=en). We have operated SACLA for more than 3000 h per year for users with a high availability beyond 92% without any significant problems.

To date, we have reported the status of SACLA in several articles. The first report of SACLA’s performance was published by Ishikawa *et al.* (2012 ▶), based on characteristics at the initial commissioning phase in 2012. More detailed descriptions of the facility and the beamline are found by Tanaka *et al.* (2015 ▶) and Tono *et al.* (2013 ▶), respectively. Now, user operations at SACLA have become much more steady and robust, and a number of scientific achievements have recently been published. In this article we provide a comprehensive overview of SACLA operating as a user facility. In §2 we summarize the recent performance of the light source and the beamline, as well as the main experimental infrastructure including optical lasers and detectors. We then review key scientific results published in 2013 and 2014, and the experimental platforms that supported these achievements. Finally, we discuss the facility upgrade to expand capabilities and capacities.

## 2. Performance of the light source and beamline   

In this section we summarize the latest status and performance of the light source, beamline optics and diagnostics, synchronized optical lasers, and detectors at SACLA.

During the three years of operation, we have developed a deep understanding of the characteristics of the SACLA light source. First, we found that the brand-new compact accelerator system is capable of producing short-wavelength XFEL light with reasonable stability and reliability. SACLA has produced XFEL light in a wide photon energy range from 4 to 20 keV, and a region between 4 and 15 keV has been routinely utilized for user operations. The typical intensity fluctuation is smaller than 10% root-mean-square (r.m.s.), and the jitter of the central photon energy is sufficiently suppressed compared with the energy bandwidth Δ*E*/*E* ≃ 0.5% full width at half-maximum (FWHM) of the SASE-XFEL light. Second, the variable-gap in-vacuum undulator gives great flexibility in the beam’s characteristics. In particular, we can produce two-colour SASE-XFEL light with a large photon-energy separation of over 30% by simply changing the magnetic gaps of the undulators in the downstream location from those in the upstream. Furthermore, we can apply a temporal delay between these pulses of up to several tens of femtoseconds with a sub-femtosecond accuracy by using a small chicane in the middle of the undulator row (Hara *et al.*, 2013*a* ▶), originally constructed for self-seeding operation with a thin diamond crystal (Geloni *et al.*, 2011 ▶; Amann *et al.*, 2012 ▶). Third, the unique injector system that combines a thermionic cathode and a velocity-bunching system generates a brilliant electron beam with a high peak current above 3 kA, which results in ultrafast duration for the XFEL pulse below 10 fs in FWHM during routine operations. Since the maximum pulse energy reaches ∼0.5 mJ, the peak power exceeds several tens of gigawatts. Typical parameters are summarized in Table 1 ▶.

To fully exploit these unique characteristics of XFEL light, one needs to employ high-quality X-ray optics, particularly for suppressing unwanted speckles and wavefront distortions under coherent illumination. For this purpose, we developed reflective mirrors (Mimura *et al.*, 2008 ▶; Yamauchi, 2015 ▶), the DCM (Ohashi *et al.*, 2013 ▶) and Be windows (Goto *et al.*, 2007 ▶) using the 1 km beamline of SPring-8, and installed them into the Optics Hutch (OH) of the SACLA beamline as key common optics for all experiments. We found that the beam profiles at the experimental station were mostly clean without noticeable speckles. The high transverse coherence property at the experimental station, which was confirmed by a speckle interferometric measurement reported by Lehmkühler *et al.* (2014 ▶), was exploited to perform coherent diffractive imaging (CDI) measurements (Takahashi *et al.*, 2013 ▶; Kimura *et al.*, 2014 ▶), as introduced in §3.4. Grating interferometry performed by Kayser *et al.* (2014 ▶) revealed that the wavefront of the reflected beam with the DCM was almost ideal, while that with plane mirrors was slightly deformed possibly due to distortion in the mirror mounting. We recently improved the mirror mounting method to reduce the effect.

X-ray focusing optics can further enhance the high intensity of XFEL light. We produced a 1 µm focusing spot with a high intensity of 10^18^ W cm^−2^ by developing a mirror system in the Kirkpatrick–Baez (KB) geometry (Yumoto *et al.*, 2013 ▶). To further increase intensity while maintaining a reasonable working distance, we needed to implement a large numerical aperture (NA), although it was restricted due to the limited distance between the undulator and the experimental station (<200 m) with a small divergence (∼microradian) of the XFEL light. To overcome this limitation, we developed a two-stage focusing system (Mimura *et al.*, 2014 ▶). Here, the first set of the KB system was utilized to enlarge the angular divergence, while the second set, with a distance of ∼70 m from the first one, installed in the experimental hutch EH5 of the SACLA–SPring-8 Experimental Facility, was used to produce a tightly focused spot. Measurements revealed that the spot size was as small as 50 nm, which corresponds to an extreme intensity of ∼10^20^ W cm^−2^. Intense XFEL pulses with these devices have enabled advanced experiments of quantum X-ray optics (Shwartz *et al.*, 2014 ▶; Tamasaku *et al.*, 2014 ▶; Yoneda *et al.*, 2014 ▶), atom, molecular and optical science (AMO) (Fukuzawa *et al.*, 2013 ▶; Tamasaku *et al.*, 2013 ▶), and elementary particle physics (Inada *et al.*, 2014 ▶), as described in §3.1. We also applied the 1 µm focused beam for conducting systematic studies on X-ray damage for optical materials including metal coating (Koyama *et al.*, 2013 ▶). Since these studies are critically important for designing XFEL beamlines, we organized an international collaboration team among the XFEL facilities, which produced beneficial results (Aquila *et al.*, 2013 ▶).

In 2013, we installed a diamond phase retarder to control the polarization of XFEL light in the photon energy range 5–20 keV (Suzuki *et al.*, 2014 ▶). We were able to convert the horizontal polarization of the monochromatic XFEL beam into vertical or circular polarization of either helicity by adjusting the angular offset of the diamond crystal from the exact Bragg condition, in a similar manner as at beamlines at synchrotron radiation facilities.

Reliable machine operation and experimental data analysis are supported by the non-destructive online diagnostic system for pulse energy, beam profile and wavelength (Tono *et al.*, 2013 ▶). Furthermore, some destructive diagnostics are useful for advanced characterization of beam properties. Absolute pulse energies were precisely calibrated using a calorimeter and a gas-monitor detector in collaboration with DESY, PTB and AIST (Kato *et al.*, 2012 ▶). For measuring the XFEL spectrum in a single shot, we developed a dispersive spectrometer that combines an elliptical mirror to increase the beam divergence, a flat silicon crystal to analyse the spectrum, and an MPCCD to detect the spectrum as a single-shot image (Yabashi *et al.*, 2006 ▶; Inubushi *et al.*, 2012 ▶). We achieved an excellent resolution of Δ*E* = 14 meV at 10 keV by using a high-order diffraction index for the distortion-free silicon analyser, which was applied to estimate the ultrafast pulse duration of XFEL light by measuring spike profiles in the SASE-XFEL spectra and comparing the results with an FEL simulation code, *SIMPLEX* (Tanaka, 2004 ▶).

A synchronized optical laser is essential for conducting time-resolved studies with a pump and probe scheme combined with XFEL pulses. We installed a Ti:sapphire (Ti:S) laser system in a laser booth in the SACLA Experimental Hall, and transported optical laser beams to every experimental station. The basic specification is given by Tono *et al.* (2013 ▶), while we recently added an amplifier to boost the pulse energy up to ∼15 mJ at a wavelength of 800 nm and a repetition rate of 60 Hz. The time resolution in pump and probe experiments is currently limited by the arrival timing jitter between XFEL and optical laser pulses. To reduce this ambiguity, an arrival timing diagnostics is critically important. LCLS successfully developed such monitors that detect the change of laser transmissivity in a thin film excited with X-rays (Harmand *et al.*, 2013 ▶). However, further reduction of X-ray pulse energy for the excitation is required for applying to SACLA, where the pulse energy is limited to sub-millijoules with a shorter wavelength option. For this purpose, we developed a scheme employing a focusing mirror to generate an intense line-focused X-ray profile, which is compatible with a spatial decoding geometry. This scheme enabled us to measure the timing with small pulse energy of 12 µJ at a photon energy of 12 keV, and to evaluate a typical timing jitter at SACLA to be 100–200 fs r.m.s. (Sato *et al.*, 2015 ▶). Based on this study, we have developed a dedicated system that can operate during user experiments, as described in §4. Note that the jitter value above was consistent with those evaluated with an independent measurement based on the THz-streaking technique performed by Juranić *et al.* (2014 ▶), which was the first demonstration of the streaking method for hard X-rays.

The combination of an XFEL light source and high-power lasers is promising for the development of high-energy-density science. Osaka University and RIKEN constructed a HERMES (High Energy density Revolution of Matter in Extreme States) system in the SACLA–SPring-8 Experimental Facility. Details are given by Kodama *et al.* (2015 ▶).

Two-dimensional detectors are critically important for exploring the full potential of XFEL light sources. At SACLA, we developed the MPCCD detectors. Based on a robust CCD technology, we were able to achieve high dynamic range, good linearity and low noise to distinguish single X-ray photons. To compensate for the relatively slow readout of the CCD arrays, we placed eight readout ports at each sensor and achieved a high frame rate of 60 Hz, which is compatible with the repetition rate of the SACLA accelerator. We constructed several variants of the camera, including a short-working-distance octal-sensor MPCCD detector (SWD-MPCCD) that covers a wide scattering angle over ±45° utilized in the DAPHNIS (diverse application platform for hard X-ray diffraction in SACLA) system (Tono *et al.*, 2015 ▶). Details of the MPCCD are given by Kameshima *et al.* (2014 ▶). We also built a data acquisition (DAQ) system to capture, analyse and store the large volume of data from the detectors, which is necessary for performing experiments efficiently and effectively, as shown by Joti *et al.* (2015 ▶).

## 3. Scientific achievements with key experimental systems   

Initial scientific results from SACLA appeared at the beginning of 2013. Since then, a number of papers in diverse fields have been published. In this section we review scientific achievements reported in 2013 and 2014 in the field of quantum X-ray optics, AMO science, elementary particle physics, ultrafast chemistry and structural biology. We then present research output with the CDI technique. We also describe the status of the key experimental systems developed for these applications. We note that the latest publication list can be found at http://xfel.riken.jp/eng/research/indexnne.html.

### 3.1. Quantum X-ray optics, AMO science and elementary particle physics   

One of the notable capabilities of SACLA is the ability to produce ultrahigh-intense X-ray pulses with the help of state-of-the-art X-ray focusing optics. This characteristic is highly useful for developing experimental investigations of quantum X-ray optics, which treats unique interactions between highly degenerated X-ray quanta with matter. Also, observation of the exotic states of atoms and molecules provides new insights into AMO science. In addition to their scientific interest, they provide firm bases for the structural determination of biological samples and nano-scale materials using intense XFEL pulses. Furthermore, the results will lead to the development of novel X-ray optical devices that can control and characterize XFEL light, such as attosecond X-ray shutters, X-ray waveguides and intensity/pulse-duration monitors.

Ueda and co-workers utilized intense X-ray pulses of 10^18^ W cm^−2^ with the 1 µm focusing system to accomplish deep inner-shell multiphoton ionization of xenon atoms. They observed highly charged ions with a charge state up to +26 using an ion time-of-flight (TOF) spectrometer (Fukuzawa *et al.*, 2013 ▶). Tamasaku *et al.* (2013 ▶) observed the generation of a hollow atom of Kr, which has double core holes in the *K*-shell, by detecting 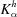 fluorescence signals with photon energies slightly higher than those of the normal *K*_α_ emissions. Here, a single core hole Kr atom created by X-ray absorption was further ionized by a succeeding X-ray photon before the atom decays to the ground state. Since the decay time is as fast as 170 as, this observation is direct proof of the high intensity of the focused XFEL pulses. As an interesting nonlinear X-ray phenomenon, Shwartz *et al.* (2014 ▶) reported observation of the second-harmonic generation (SHG) for 7.3 keV photons by using focused monochromatic XFEL pulses. The periodic nature of the electron density in a diamond crystal was utilized to satisfy a phase-matching condition, which helped with the observation of the nonlinear phenomenon with a moderate X-ray intensity of ∼10^16^ W cm^−2^.

A further increase of X-ray intensity enhances the capability to investigate various nonlinear X-ray effects. For this purpose, the two-stage 50 nm focusing system for producing an ultrahigh intensity of 10^20^ W cm^−2^ was utilized. Yoneda *et al.* (2014 ▶) observed an increase in the transmissivity of 7.1 keV X-rays by an order of magnitude for a 20 µm iron foil. This is the first observation of saturable absorption of hard X-rays, which originated from the depletion of atoms in the ground state after photoionization of the *K*-shell electrons. The wavefront of the transmitted X-ray beam was modulated with an intensity-dependent complex refractive index. Tamasaku *et al.* (2014 ▶) demonstrated two-photon absorption of 5.6 keV X-rays, which is the first observation of the third-order nonlinear effect of X-rays, at the Ge *K*-edge (11.1 keV) by detecting the Ge *K*_α_ emissions.

Intense XFEL pulses from SACLA can reveal nonlinear interactions with not only materials but also with vacuum. Quantum electrodynamics (QED) predicts the existence of photon–photon (γ–γ) scattering processes as a nonlinear effect of vacuums. An advantage of short-wavelength X-rays is a higher cross section of γ–γ scattering, which is proportional to the sixth power of the photon energy. The cross section for 6.5 keV X-rays becomes 23 orders of magnitude larger than that for 1 eV visible light. Asai and co-workers first attempted a direct search for γ–γ scattering in the X-ray region (Inada *et al.*, 2014 ▶). To achieve collision between dual X-ray beams, a beam-splitter optics based on an X-ray interferometric technique was developed. Even though no events were observed in the signal region (18.1–19.9 keV), the cross section of γ–γ scattering was determined to be smaller than 1.7 × 10^−24^ m^2^ at a 95% confidence level. Further improvement of monochromaticity, as well as enhancement of the intensity, will enable us to improve the detection limit by several orders of magnitudes and to approach a prediction of QED.

### 3.2. Ultrafast chemistry   

Ultrafast chemistry is one of the most promising fields developed with XFELs. By probing X-ray absorption, emission, scattering and diffraction signals, one can investigate the ultrafast changes of electronic states and structures in molecular systems stimulated by external optical pumps. SACLA offers an ultrafast pulse duration, high stability and short-wavelength capability below 1 Å, which provides an excellent opportunity for conducting these experiments with high accuracy. As an initiative in this field, we have developed a new method for femtosecond X-ray absorption spectroscopy.

Since SASE-XFEL light has a finite bandwidth of the order of Δ*E*/*E* ≃ 10^−3^, one can design X-ray absorption spectroscopy in a dispersive geometry so as to detect the whole spectrum in a single shot (Yabashi *et al.*, 2006 ▶; Inubushi *et al.*, 2012 ▶). This scheme is much more efficient and robust compared with the wavelength-scanning method. However, the stochastic nature of SASE-XFEL pulses results in shot-to-shot changes in the spectrum, which significantly degrades the data quality with a conventional dispersive geometry. To address this problem, we developed a new scheme that combines a dispersive X-ray spectrometer with a transmission grating to generate multiple branches. We inserted a sample in the 1^st^ order branch, while we utilized the −1^st^ order branch for normalization of the spectrum. Following confirmation of the feasibility of using a static Zn foil (Katayama *et al.*, 2013 ▶), we conducted time-resolved measurements for a liquid beam of aqueous ammonium iron(III) oxalate solution excited with a 400 nm femtosecond laser in collaboration with Kyoto University and Tokyo University of Agricultural and Technology (Obara *et al.*, 2014 ▶). We successfully detected a small change in the absorbance induced by the pump of the order of 10^−3^. The photoexcited iron complex showed a red shift for the iron *K*-edge in an ultrafast time constant of 260 fs. Although the time resolution in this study was limited by the arrival timing jitter between the XFEL and the optical laser pulses, it will be improved by introducing an arrival timing monitor, as shown in §2 and §4.

Recently, Ihee, Adachi and co-workers reported a time-resolved wide-angle scattering (WAXS) experiment for investigating bond-making dynamics in solution (Kim *et al.*, 2015 ▶). They first succeeded in providing direct visualization of the bond formation process in a gold trimer complex [Au(CN)_2_^−^]_3_ after photoexcitation in sub-picosecond and sub-angstrom resolutions.

### 3.3. Structural biology   

The unique characteristics of XFEL light are particularly useful for developments of structural biology. The capability of single-shot detection of diffraction signals using a femtosecond X-ray pulse enables one to obtain intrinsic structures that are free from structural damage caused by the radicals and reactants produced at X-ray irradiation. One can analyse the structures of micro- or nano-sized protein crystals in physiological conditions. Furthermore, the combination of a pump–probe technique enables investigations of the intermediate states of molecules during ultrafast reactions at an atomic resolution.

At SACLA, the first experiment in this field was performed in 2012 by an international collaboration team, which successfully detected weak anomalous signals from sulfur atoms naturally included in a protein (Barends *et al.*, 2013 ▶). This study employed a serial femtosecond crystallography (SFX) scheme (Boutet *et al.*, 2012 ▶) using an experimental chamber developed by Tohoku University and Kyoto University, modelled after a CAMP chamber (Strüder *et al.*, 2010 ▶). Recently, we developed an original experimental platform, DAPHNIS, for SFX experiments (Tono *et al.*, 2015 ▶). DAPHNIS is composed of a compact sample chamber, sample injectors and a SWD-MPCCD detector. The sample chamber is filled with helium, instead of vacuum for the MAXIC or the CAMP, which substantially facilitates operation of sample injectors while precisely controlling temperature and humidity. We are able to attach various types of sample injectors, including a grease-carrier injector recently developed by Sugahara *et al.* (2015 ▶) for efficient sample delivery. The detector with a large-diameter Be window was completely separated from the chamber so as to avoid contamination by samples or damage resulting from the optical pump lasers. DAPHNIS is thus applicable to perform WAXS measurements combined with the pump–probe technique.

As an alternative scheme, a conventional diffractometer system utilized at SPring-8 was introduced at SACLA for performing damage-free structural analysis on large-sized crystals. To measure diffraction signals from a fresh part, the sample is translated by using stages in each shot. Ago *et al.* confirmed the feasibility of this scheme by performing structural analysis of bovine cytochrome c oxidase, a large (420 kDa) highly radiation-sensitive membrane protein with a resolution of 1.9 Å (Hirata *et al.*, 2014 ▶). Furthermore, Shen *et al.* recently applied this system to reveal the intrinsic structure of photosystem II (PS-II) at an excellent resolution of 1.95 Å (Suga *et al.*, 2014 ▶).

### 3.4. Coherent diffractive imaging   

SACLA’s excellent transverse coherence with high brilliance is useful for performing CDI experiments for non-periodic nano-sized particles. Two experimental chambers with vacuum compatibility have been constructed at SACLA. One is a multiple-application X-ray imaging (MAXIC) chamber (Song *et al.*, 2014 ▶). Here we designed the chamber for versatile purposes. The other is a KOTOBUKI-I chamber that can accommodate a cryogenic sample environment, developed by Keio University (Nakasako *et al.*, 2013 ▶). In both cases, most samples are mounted on a thin membrane or confined in a special container (Kimura *et al.*, 2014 ▶), and translated using stages to avoid the exposures of multiple shots. This sample delivery system is different from those for SFX where liquid injectors are fully employed (see §3.3), because parasitic X-ray scattering from the liquid jet, which increases the background especially in a small-angle region, is not appropriate for CDI analysis. For detectors, one can combine an octal-sensor MPCCD detector for covering a wide-angle region and a dual-sensor MPCCD for a small-angle region set in tandem. The 1 µm focusing system is utilized for increasing the intensity at the sample. A typical spatial resolution under the present experimental conditions is a few tens of nanometres for biological samples, while it is improved to several nanometres for inorganic samples composed of heavy elements.

Takahashi *et al.* (2013 ▶) measured coherent diffraction patterns for Au/Ag nano-boxes and Ag nano-cubes with sizes of ∼150 nm, and reconstructed two-dimensional projections of electron density with sub-10 nm resolution from single-shot images. This enabled statistical analysis of particle sizes, external shapes and internal structures from a large number of samples. Miao *et al.* reconstructed the three-dimensional structure of gold nanocrystals based on the highly symmetric nature of the samples (Xu *et al.*, 2014 ▶).

For biological targets, Nishino *et al.* reconstructed whole-cell images of *M. lacticum* in a living state, contained in a micro-liquid enclosure without any stains, with a full-period spatial resolution of ∼37 nm (Kimura *et al.*, 2014 ▶). They observed a high electron density region inside the cell, which possibly related to condensation of the DNA molecules. Song *et al.* analysed the nanostructure formation of RNA interference (RNAi) microsponges by combining single-shot imaging at SACLA and tomographic correlative measurements at SPring-8 (Gallagher-Jones *et al.*, 2014 ▶). They found that the RNAi microsponges contain high electron density cores, which provides useful information for designing a system for efficient drug delivery.

In the Bragg diffraction geometry, Newton *et al.* (2014 ▶) performed a time-resolved CDI measurement for a single vanadium dioxide nanocrystal mounted on a diffractometer. Structural changes induced by 800 nm laser irradiation were traced at a picosecond temporal resolution.

## 4. Perspective on facility upgrade   

The SACLA facility has the capability of operating five beamlines simultaneously with independent undulators. At the inauguration in 2012, we started with two beamlines: BL3 for hard X-ray FEL and BL1 for wide-range spontaneous emissions. Since then, experiments in broad fields have been conducted at BL3. These diverse studies become possible due to the flexible design of the experimental stations, which are equipped with only selected beamline components while providing a lot of space for introducing various devices, rather than setting dedicated instruments for specific applications, to address the requirement in the initial start-up phase.

Although results from experiments are substantial, the limited availability of beam time, a common concern for the world’s XFEL activities, has become an increasingly serious problem. To address this issue, we started construction of a new beamline BL2 in 2013 as the second hard X-ray FEL beamline at SACLA. The undulator specification, as well as the beamline configuration in the OH, are basically the same as those for BL3.

**Figure 1:**
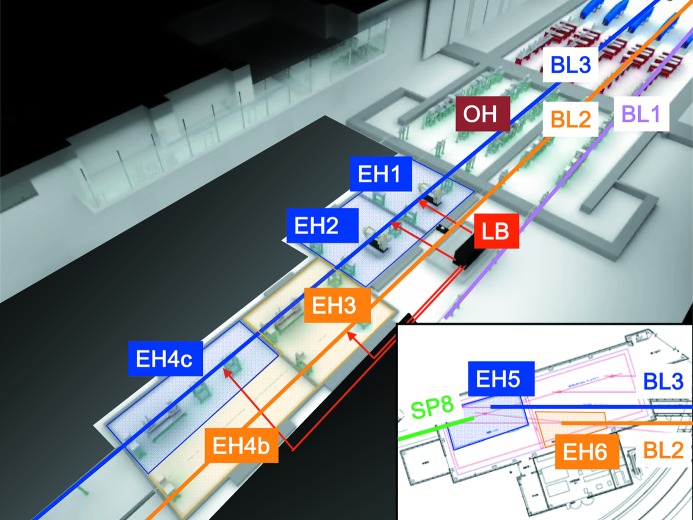
A schematic view of the SACLA experimental hall. EH1 (advanced diagnostics), EH2 (unfocused), EH4c (1 µm focusing) and EH5 are attributed to BL3, while EH3 (1 µm focusing), EH4b (SAXS detectors) and EH6 are dedicated to BL2. Femtosecond optical lasers are independently delivered from the laser booth (LB) to BL3 (EH1, EH2, EH4c) and BL2 (EH3). The inset shows a schematic of the SACLA–SPring-8 Experimental Facility, which is equipped with the high-power laser facility HERMES.

Fig. 1 ▶ shows a schematic of the SACLA experimental hall with a revised beamline configuration in the autumn of 2014. The former EH4 was divided into two hutches, EH4b and EH4c, and we assigned EH3 and EH4c for BL2. The main instrument is installed in EH3 that is equipped with an improved version of the 1 µm focusing system. For the new focusing system, we employ Rh-coated mirrors with increased lengths from 400 mm to 600 mm. We can choose the mirror incident angles of either ∼3.7 mrad or ∼2.0 mrad by translating the mirrors to be operated. In EH4b, one can set up detectors for measuring small-angle X-ray signals, if required. Femtosecond optical lasers, which are similar to those utilized at BL3, are available. At BL2, we plan to fully exploit experimental platforms, for example the DAPHNIS platform for SFX and a MAXIC system for CDI, as introduced in §3. We install these instruments for a relatively long term so as to perform series of experiments without changing the setup. These experimental platforms will improve the efficiency of typical experiments and mitigate the hurdles for performing experiments at SACLA for a broad range of researchers, including industrial users and non-experts of XFEL experiments. Also, we plan to extend BL2 to EH6 in the SACLA–SPring-8 Experimental Facility for conducting high-energy-density science with the HERMES system.

We started the commissioning of BL2 in the autumn of 2014, and observed the first lasing in October. We plan to commence user operations in 2015, which will mean the start of operations for multiple hard X-ray FEL beamlines. A switching magnet located at the upstream of the undulators is used to select the operational beamline to which the electron beam is transported. Since the SACLA accelerator system allows us to change the electron beam energy pulse-to-pulse operated at a repetition rate of 60 Hz, we are able to operate multiple beamlines simultaneously with a single accelerator (Hara *et al.*, 2013*b* ▶). This expansion of experimental capacities at SACLA will contribute significantly to the development of many fields of science. Furthermore, the on-demand electron-beam delivery system will enable us to employ the SACLA linac for an injector that is compatible with the upgraded ultralow-emittance SPring-8 storage ring that is under planning (Tanaka, 2014 ▶).

In contrast to the dedicated experimental setups in BL2, we continue to operate BL3 for advanced experiments in broad fields while maintaining greater flexibility and versatility with enhanced capabilities. In the summer of 2014, we rearranged the configuration of the experimental hutches so as to be compatible with operation of BL2. Currently, EH1, EH2 and EH4c are allocated to BL3, as shown in Fig. 1 ▶. In the OH, we replaced the beamline Si mirrors of length *L* = 400 mm with new ones with *L* = 500 mm that are partially coated with Rh for enabling optional utilization of the pink beam in a higher photon energy region above 20 keV. We also installed a transmission grating developed by Paul Scherrer Institute into the OH, which enables non-destructive diagnostics of the single-shot spectrum and the arrival timing between the XFEL and optical lasers pulses in combination with a new chamber installed in EH1. We installed the improved 1 µm focusing system in EH4c, similar to EH3 of BL2. We observed that the beamline transmissivity of 10 keV XFEL light to samples was improved from ∼50% to 78% at 10 keV.

In the autumn of 2013, we constructed a self-seeding system in the undulator section of BL3, and observed a seeded signal for 10 keV X-rays. We continue commissioning to provide stable seeded XFEL light for user operation.

Finally, we plan to relocate the decommissioned SCSS accelerator to the SACLA undulator hall. By connecting it to the undulator of BL1, we will be able to operate a FEL light source in the EUV to soft X-ray region. Since the new accelerator (SCSS+) is operated independently of the SACLA main linac, we expect a substantial increase of availability in the softer X-ray region.

## Figures and Tables

**Table 1:** Typical parameters for the accelerator and BL3 of SACLA, based on Schmser *et al.* (2014 ▶) with updates

Electron beam energy	5.18.5GeV
Peak current	>3kA
Bunch charge	0.20.3nC
Repetition rate	30Hz (60Hz maximum)
Bunch duration	20fs
Normalized slice emittance	0.4m
Photon energy	4.020 keV
Saturation power	660 GW
FEL pulse energy	0.5mJ at 10keV
Photon pulse duration	210fs
Undulator period	18mm
Undulator parameter	2.2
